# The combination of high glucose and LPS induces autophagy in bovine kidney epithelial cells via the Notch3/mTOR signaling pathway

**DOI:** 10.1186/s12917-022-03395-1

**Published:** 2022-08-11

**Authors:** Yaocheng Cui, Hongrui Guo, Qin Zhang, Jing Fang, Yue Xie, Shiyi Chen, Xiaoping Ma, Liping Gou, Hengmin Cui, Yi Geng, Gang Ye, Zhijun Zhong, Zhihua Ren, Ya Wang, Junliang Deng, Shuming Yu, Suizhong Cao, Zhisheng Wang, Zhicai Zuo

**Affiliations:** 1 Key Laboratory of Animal Disease and Human Health of Sichuan Province, College of Veterinary Medicine, Sichuan Agricultural University, Chengdu, 611130 Sichuan China; 2 Chengdu Customs of the People’s Republic of China, Chengdu, 610095 Sichuan China; 3 College of Animal Science and Technology, Sichuan Agricultural University, Chengdu, 611130 Sichuan China; 4 Animal Nutrition Institute, Sichuan Agricultural University, Chengdu, 611130 Sichuan China

**Keywords:** High glucose, LPS, Autophagy, Notch3, mTOR

## Abstract

**Background:**

Aside respiratory diseases, beef cattle may also suffer from serious kidney diseases after transportation. Hyperglycemia and gram-negative bacterial infection may be the main reasons why bovine is prone to severe kidney disease during transportation stress, however, the precise mechanism is still unclear. The purpose of the current study is to explore whether the combined treatment of high glucose (HG) and lipopolysaccharide (LPS) could induce madin-darby bovine kidney (MDBK) cells injury and autophagy, as well as investigate the potential molecular mechanisms involved.

**Results:**

As we discovered, the combined effect of HG and LPS decreased MDBK cells viability. And, HG and LPS combination also induced autophagy in MDBK cells, which was characterized by increasing the expression of LC3-II/I and Beclin1 and decreasing p62 expression. LC3 fluorescence signal formation was also significantly increased by HG and LPS combination treatment. Furthermore, we measured whether the mammalian target of rapamycin (mTOR) and the Notch3 signaling pathways were involved in HG and LPS-induced autophagy. The results showed that the combination of HG and LPS significantly increased the protein expression of Notch3 and decreased protein expression of p-mTOR, indicating that Notch3 and mTOR signaling pathways were activated. However, co-treatment with the Notch3 inhibitor (DAPT) could reverse the induction of autophagy, and increased the protein expression of p-mTOR.

**Conclusions:**

This study demonstrated that the combination effect of HG and LPS could induce autophagy in MDBK cells, and the Notch3/mTOR signaling pathway was involved in HG and LPS-induced autophagy.

**Supplementary Information:**

The online version contains supplementary material available at 10.1186/s12917-022-03395-1.

## Introduction

The cattle industry loses 1 billion USD annually from transportation stress in the United States [1]. Transportation stress could significantly increase the concentration of glycemia in beef cattle [2–4]. Several studies have demonstrated that hyperglycemia causes various diseases such as stress hyperglycemia and diabetes in humans and animals [5–7]. Recently, it is not clear the harmful of hyperglycemia in beef cattle is unfavorable to beef cattle. Hyperglycemia has been reported to cause damage to multiple organs and tissues, such as the kidney, heart, and cardiovascular system [8]. Transportation stress also could inhibit the bovine immune system, thereby increasing the risk of gram-negative bacterial infection [1, 9]. *Pasteurella multocida*, *Mannheimia haemolytica*, and *Escherichia coli* have been reported to trigger not only respiratory disease but also sepsis in cattle [10–13]. The kidney is one of the most vulnerable organs to hyperglycemia and sepsis [14, 15]. Hence, the kidney may suffer from hyperglycemia and LPS toxicity during transportation stress. However, the mechanism of the combined action of hyperglycemia and LPS on bovine kidneys is still unclear.

Hyperglycemia triggers stress hyperglycemia and serious diabetic kidney disease in humans, mice, and rats [14, 16–18]. LPS, as a kind of endotoxin produced by gram-negative bacteria, could induce septic acute kidney injury in humans, mice, and rats [19–21]. Existing studies have demonstrated the induction of oxidative stress, inflammation, apoptosis, and pyroptosis in diabetic kidney disease or septic acute kidney injury [20, 22–24]. Recent studies have also found that autophagy plays a vital role in diabetic kidney disease or septic acute kidney injury [25, 26]. It has been reported that HG inhibits cranial neural crest survival by activating autophagy in the chick embryo [27]. Autophagy is activated by the lactate/SIRT3/AMPK pathway in septic mice and in LPS‑treated HK‑2 cells [28]. Cell death has been reported to be mediated possibly by uncontrolled or overstimulated autophagy [29]. Therefore, autophagy may remove protein aggregates and damaged or redundant organelles to maintain cell homeostasis and cell integrity [30], thereby regulating nephropathy [31, 32]. Interestingly, it has been reported that serum LPS concentration is related to the progression of renal disease in Finnish patients with type 1 diabetes [33]. Regrettably, the combined effect of HG and LPS on autophagy in renal cells remains unclear.

Regulation of autophagy is an extremely complex process involving many signaling pathways [30]. mTOR signaling, one of the main signal pathways regulating autophagy, is a pivotal regulator of autophagy by regulating many aspects of the autophagy process, such as initiation, process, and termination [34]. Notch receptors are highly conserved signaling molecules that are widely distributed on the surface of a variety of cells [35]. In mammals, there are four Notch receptors namely, Notch1, Notch2, Notch3 and Notch4 [36]. Recent studies have shown that Notch3 is abnormally activated, plays crucial roles in nephropathy, and directly affects the prognosis as well as the outcome of nephropathy [37–39]. Moreover, Notch3 has also been found to play an important regulatory role in autophagy [40–42]. Previous study have reported that mTOR is the major up-stream regulator of Notch3 [43], however, the latest research found that Notch3 may be the major up-stream regulator of the mTOR signaling [44]. However, whether Notch3 has a regulatory effect on mTOR activity requires further investigation.

Taken together, the purpose of the current study is to explore whether the combined treatment of HG and LPS could induce MDBK cells injury, meanwhile, the autophagy and its potential molecular mechanisms induced by HG and LPS are evaluated, including mTOR and Notch3 signaling pathways.

## Materials and methods

### Cell, chemical and reagents

MDBK cells were purchased from China Center for Type Culture Collection (CCTCC). DMEM/F12(HAM) medium, DMEM low glucose medium, and DMEM high glucose medium were purchased from Biological Industries, Israel. LPS from *Escherichia coli 055: B5* was purchased from Solarbio, China. Notch3 inhibitor (DAPT) was purchased from Selleck, America.

### Cell culture

MDBK cells were cultured in DMEM/F12 (HAM) medium containing 10% fetal bovine serum (Gibco), 100 U/ml penicillin, and 100 ug/ml streptomycin (Solarbio). MDBK cells were then incubated at 37 ℃ and 5% CO_2_. When the density of MDBK cells reached 50–60% in T25 cell bottle, MDBK cells were cultured and incubated at 37 ℃ and 5% CO_2_ in DMEM medium containing 5.5 mM glucose and 25.5 mM glucose with different concentrations of LPS (0, 1, 2 and 5 μg/ml), respectively.

### Cell counting kit-8 (CCK-8) assay for cell growth

Trypsinized MDBK cells (2 × 10^5^ cells) were resuspended in complete DMEM/F12 (HAM) medium, seeded in a 96-well plate, and then incubated with 0, 1, 2 and 5 μg/ml of LPS in the previously mentioned normal and high-glucose conditions under 37 °C, 5% CO_2_. Cell proliferation was evaluated by CCK-8 assay following the manufacturer’s instructions after 24 h. All experiments were performed in triplicate.

### Quantitative real-time PCR (qRT-PCR) analysis

Primers for qRT-PCR were designed using Primer Premier Software 5.0 (Premier Bio Soft International). The sequences are shown in Table 1. The qRT-PCR was performed in a 20 μL reaction per well in a 96-well plate containing 3 μL diluted cDNA, 10 μL SYBR® Premix Ex Taq II (Tli RNASEH Plus), 0.4 μL each upstream and downstream primers (10 μM), and 6.2 μL ddH2O. The qRT-PCR amplification procedure was as follows: 94 ℃ 30 s; 94 ℃ 5 s, 60 ℃ 15 s, 72 ℃ 10 s, 39 Cycles; Melt Curve: 65℃ → 95℃. The qRT-PCR reactions were performed on the CFX96 Quantitative Real-time PCR system (Bio-Rad) using qRT-PCR Kit (Takara). Melting curve analysis verified the reliability of each qRT-PCR reaction. Quantitative measurements were determined by using the 2^−ΔΔCt^ method, and the mRNA expression of β-actin gene was used as the internal control.

**Table 1:** The primers used for Real-time PCR

Name	Primer sequence (5’-3’)
Notch3	Forward: CAGACACCAATGCCCAGGAC
	Reverse: CAGGTCTGTAGAGCGGTTCC
mTOR	Forward: GCTGGCACTTGCTCACAAAA
	Reverse: GAAGGCATCAATCTTGCGGG
LC3B	Forward: TGCCGTCCGAGAAAACCTTCAAAC
	Reverse: CGGGATTTTGGTAGGATGCTGCTC
p62	Forward: CTGGGAGATGGGCACACC
	Reverse: TGGGATCTTCCGATGGACCA
Beclin1	Forward: TCCATTACTTGCCACAGCC
	Reverse: GCCATCAGATGCCTCCC
β-actin	Forward: CGTCCGTGACATCAAGGAGAAGC
	Reverse: GGAACCGCTCATTGCCGATGG

### Western blot analysis

MDBK cells were lysed by radio immunoprecipitation assay (RIPA, Solarbio) lysis buffer with phenylmethylsulfonyl fluoride (PMSF, MCE). The concentration of proteins was measured using a bicinchoninic acid (BCA, Thermo Fisher Scientific) protein assay kit. The proteins were separated by SDS polyacrylamide gel electrophoresis (SDS-PAGE) and were transferred to polyvinylidenedifluoride (PVDF) membranes. The membranes were blocked in a nonfat dry milk solution for 30 min. Then, the membranes were incubated overnight at 4 ℃ with Notch3 (affinity, 1:1000 dilution), anti-mTOR (Bioss, bs-1992R, 1:1000 dilution), anti-p-mTOR (Affinity, AF3308, 1:1000 dilution), anti-LC3A (Novus Biologicals, NB100-2331, 1:1000 dilution), anti-p62 (GeneTex, GTX100685, 1:1000 dilution) and anti-Beclin1 (CST, 3495 T, 1:1000 dilution) antibody primary antibodies in dilution buffer. After washing with TBS-T, the membranes were incubated with horseradish peroxidase (HRP)-conjugated anti-rabbit (Solarbio, SE134, 1:3000 dilution) at room temperature for 30 min. The membranes were developed using the ECL Western blot system (Tanon-5200) according to the manufacturer’ s instruction.

### Immunofluorescence analysis

MDBK cells were seeded in 24 mm glass-bottomed microwell dishes. After was cultured according to the description mentioned above, MDBK cells were then washed with PBS three times and fixed with 4% paraformaldehyde (Servicebio) for 15 min. Next, 0.1% TritonX-100 (Servicebio) was used for cell permeabilization for 20 min. The cells were blocked with 3% BSA (Servicebio) in PBS at room temperature for 30 min and then were incubated with anti-LC3A (Novus Biologicals, NB100-2331, 1:100) overnight at 4℃. The next day, the cells were incubated with CY3 (Servicebio, GB21303, 1:300) at room temperature for 1h. The 4,6-diamidino-2-phenylindole (DAPI, Servicebio) counterstain was used to show the MDBK cells nuclei. Finally, the stained MDBK cells were observed by immunofluorescence microscopy (NIKON ECLIPSE C1).

### Transmission electron microscopy (TEM) analysis

MDBK cells were cultured as described above. MDBK cells were washed with PBS three times and collected by a cell scraper. Then, the cells were centrifuged at 1000 g for 10 min. Precipitated MDBK cells were fixed with ice-cold 3% glutaraldehyde (Solarbio) and then post-fixed in 1% osmium tetroxide (Leica). The cell clumps were cut into ultrathin sections after they were embedded in Epon epoxy resin (Chengdu Rongshengke Biotechnology Co., Ltd). The ultrathin sections were stained with 0.1% lead citrate (Chengdu Rongshengke Biotechnology Co., Ltd) and 10% uranyl acetate (Chengdu Rongshengke Biotechnology Co., Ltd) and were observed with a transmission electron microscope (JEM-1400PLUS, JEOL).

### Statistical analyses

All the experiments were performed in triplicates. The data were presented as the mean ± SD and were analyzed using variance SPSS 24.0. The differences in the means were determined by a one-way analysis of variance (ANOVA) for multiple comparisons followed by the least significant difference (LSD) test for two-group comparisons among the multiple comparisons. *P*-value of less than 0.05 was considered statistically significant (# or * *P* < 0.05) and *P*-value of less than 0.05 was considered extremely significant (# or ** *P* < 0.01).

## Results

### The combined treatment of HG and LPS induced autophagy in MDBK cells

**Fig. 1:**
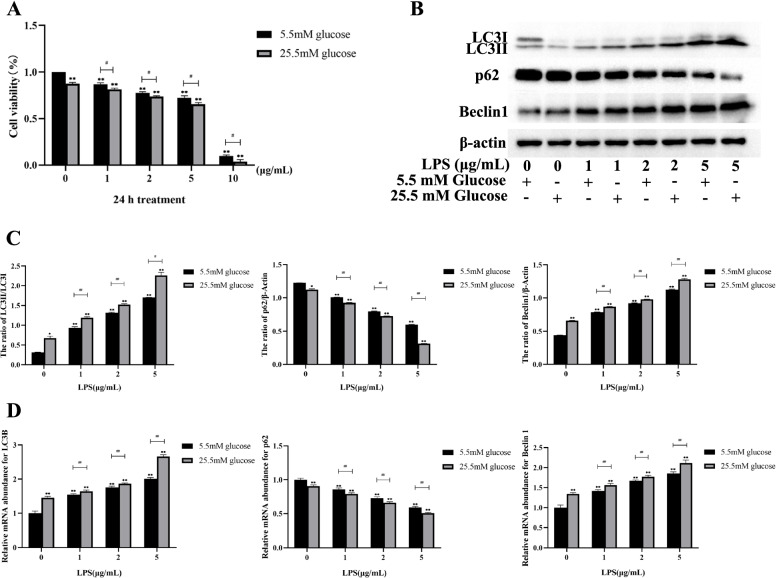
HYPERLINK "sps:id::fig1||locator::gr1||MediaObject::0" Co-treatment of HG and LPS induced autophagy in MDBK cells. Note: 0, 1, 2, 5 μg/mL LPS are co-treated with 5.5 mM glucose and 25.5 mM glucose respectively for 24 h in MDBK cells. **A** The cell viability; **B**-**C** The protein level of LC3-II/I, p62 and Beclin1; **D** The mRNA expression level of LC3B, p62 and Beclin1. *0.01 < *P* < 0.05 and ** *P* < 0.01 compared with control group; #:0.01 < *P* < 0.05; ##: *P* < 0.01

To evaluate the combined effect of HG and LPS on MDBK cells, 0, 1, 2, and 5 μg/mL LPS were co-treated with 5.5 mM glucose or 25.5 mM glucose for 24 h in MDBK cells, and the cell proliferation was examined. The results showed that compared with the 5.5 mM glucose and the same concentration of LPS co-treated groups, the MDBK cells viability of the 25.5 mM glucose and 1 μg/mL LPS, 25.5 mM glucose and 2 μg/mL LPS, and 25.5 mM glucose and 5 μg/mL LPS treatment groups decreased significantly (*P* < 0.05) (Fig. 1A). In addition, the combination of 10 μg/mL LPS with 5.5 mM glucose or 25.5 mM glucose caused an excessive decrease in cell viability. Based on the above observations, LPS at concentrations (0, 1, 2, 5 μg/mL) co-treated with 5.5 mM or 25.5 mM glucose respectively were chosen for each subsequent experiment.

Next, to explore the combined effect of HG (at concentrations 5.5 mM and 25.5 mM) and different concentrations of LPS (0, 1, 2, and 5 μg/ml) on autophagy in MDBK cells, the protein and mRNA levels of LC3, p62 and Beclin1 in MDBK cells were detected by Western blot and qRT-PCR, and the intensity of red fluorescent labeled LC3 was detected by immunofluorescence assay. Figure 1B-C illustrated that, compared with the MDBK cells co-treated with the 5.5 mM glucose and 1 μg/mL LPS, 5.5 mM glucose and 2 μg/mL LPS, and 5.5 mM glucose and 5 μg/mL LPS, the 25.5 mM glucose combination with the same concentration of LPS (1, 2 and 5 μg/mL) treatment groups showed a significant increase in the protein levels of LC3-II/I and Beclin1 (*P* < 0.05) whereas the protein level of p62 was decreased significantly (*P* < 0.05).Consistent with the protein experiment results, relative to the 5.5 mM glucose and 1 μg/mL LPS, 5.5 mM glucose and 2 μg/mL LPS, and 5.5 mM glucose and 5 μg/mL LPS treatment groups (Fig. 1D), the mRNA expression level LC3B and Beclin1 were increased significantly (*P* < 0.01), whereas the mRNA expression level p62 was decreased significantly (*P* < 0.01) in the 25.5 mM glucose and the same concentration (1, 2 and 5 μg/mL) of LPS treatment groups. Moreover, compared with other groups, the protein and mRNA expression levels of the 25.5 mM glucose and 5 μg/mL LPS treatment group were the most significant.

**Fig. 2:**
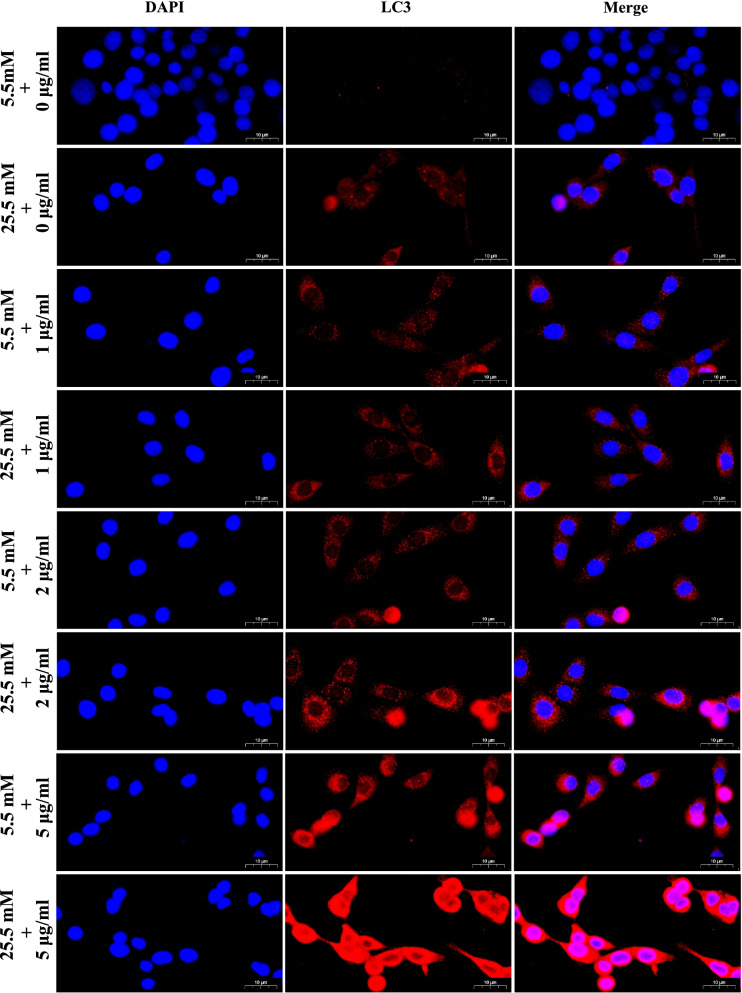
Immunofluorescence observation of LC3 in MDBK cells after HG and LPS co-stimulation. Note: 0, 1, 2, 5 μg/mL LPS are co-treated with 5.5 mM glucose and 25.5 mM glucose respectively for 24 h in MDBK cells. LC3 was labeled with red fluorescence by Cy3, and the nucleus was labeled with blue fluorescence by DAPI. Scale bar, 10 µm

Furthermore, immunofluorescence assay was used for measuring protein levels of LC3 in the MDBK cells in the present study. As shown in Fig. 2, compared with MDBK cells co-treated with the 5.5 mM glucose and LPS concentration (1, 2, and 5 μg/mL) respectively, the 25.5 mM glucose and 1 μg/mL LPS, 25.5 mM glucose and 2 μg/mL LPS, and 25.5 mM glucose and 5 μg/mL LPS treatment groups showed a significant increase in the intensity of red fluorescent labeled LC3. Moreover, compared with other groups, the intensity of red fluorescent labeled LC3 of the 25.5 mM glucose and 5 μg/mL LPS treatment group was the most obvious.

To investigate whether the combined treatment of HG and LPS induces autophagic flux in MDBK cells, chloroquine (CQ) was used. As shown in Figure S 2, incubation of cells with CQ resulted in significantly increased LC3-II expression (*P* < 0.01), and treatment with HG + LPS + CQ revealed a further increase in LC3-II expression (*P* < 0.01).

In summary, these findings indicated that co-treatment of HG and LPS induced autophagy in MDBK cells.

### Co-treatment of HG and LPS induced Notch3 and mTOR signaling pathways in MDBK cells

**Fig. 3:**
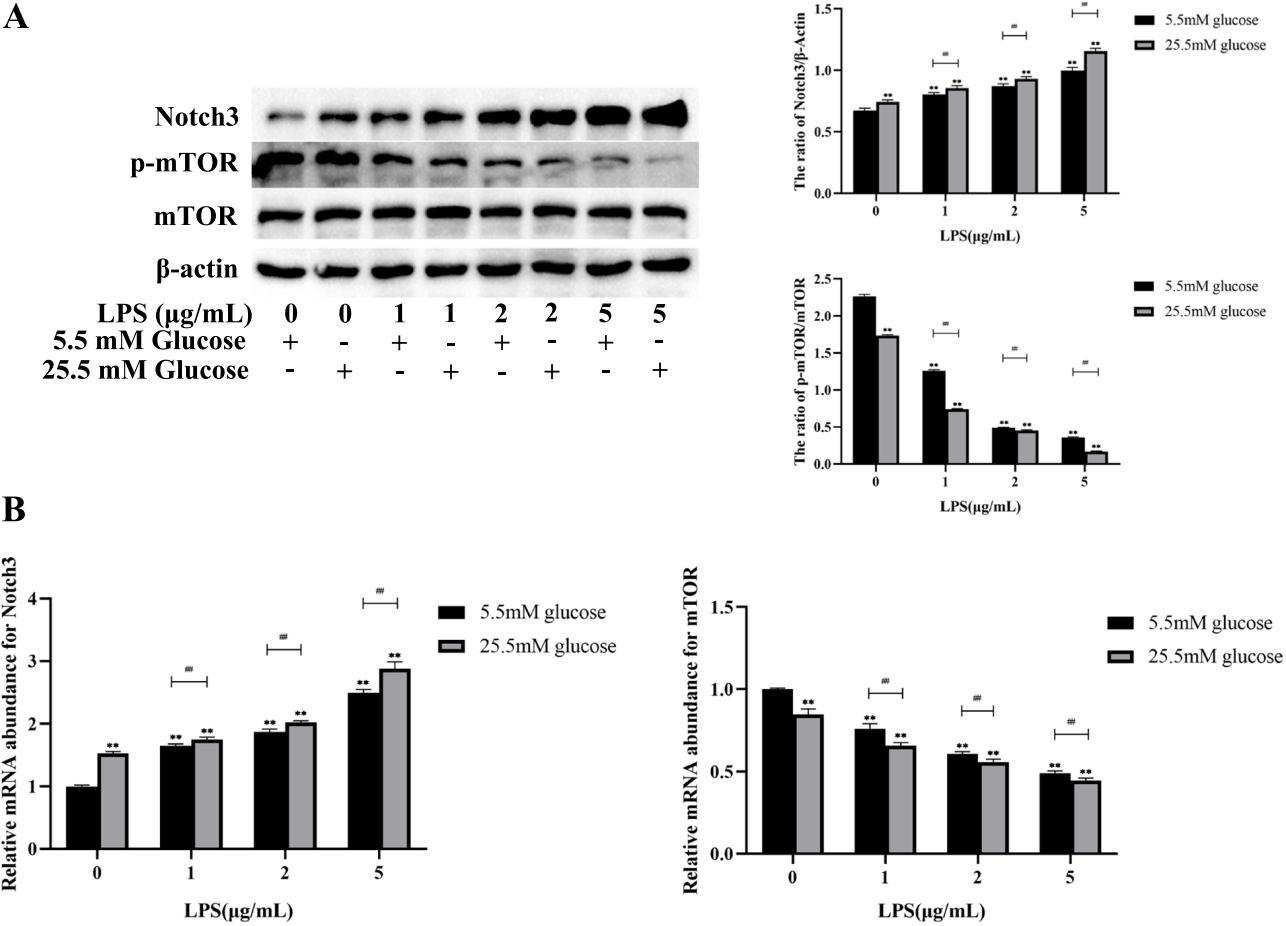
Co-treatment of HG and LPS induced Notch3 and mTOR signaling pathways in MDBK cells. Note: 0, 1, 2, 5 μg/mL LPS are co-treated with 5.5 mM glucose and 25.5 mM glucose respectively for 24 h in MDBK cells. **A** The protein level of Notch3, mTOR and p-mTOR; **B** The mRNA expression level of Notch3 and mTOR. *0.01 < *P* < 0.05 and ** *P* < 0.01 compared with control group; #:0.01 < *P* < 0.05; ##: *P* < 0.01

To investigate the role of Notch3 and mTOR in MDBK cells induced by the combination of HG and LPS, the protein and mRNA expression levels of Notch3 and mTOR in MDBK cells were detected by western blot and qRT-PCR (Fig. 3A-B). Figure 3A showed that compared with the MDBK cells co-treated with the 5.5 mM glucose and 1 μg/mL LPS, 5.5 mM glucose and 2 μg/mL LPS, and 5.5 mM glucose and 5 μg/mL LPS, the 25.5 mM glucose and the same concentration of LPS treatment groups showed a significant increase in the protein levels of Notch3 (*P* < 0.01) whereas the protein level of p-mTOR was decreased significantly (*P* < 0.01). These results were consistent with the mRNA experiment results, compared with the 5.5 mM glucose and 1 μg/mL LPS, 5.5 mM glucose and 2 μg/mL LPS, and 5.5 mM glucose and 5 μg/mL LPS treatment groups, the 25.5 mM glucose and the same concentration of LPS treatment groups showed a significant increase (*P* < 0.01) in the mRNA expression levels of Notch3 and a significant decrease (*P* < 0.01) in mRNA expression levels of mTOR (Fig. 3B). Moreover, compared with other groups, the protein and mRNA expression levels of the 25.5 mM glucose and 5 μg/mL LPS treatment group were the most significant.

These results revealed that co-treatment of HG and LPS induced Notch3 and mTOR signaling pathways in MDBK cells.

### Notch3-mediated mTOR signaling pathway was involved in HG and LPS-induced autophagy in MDBK cells

**Fig. 4:**
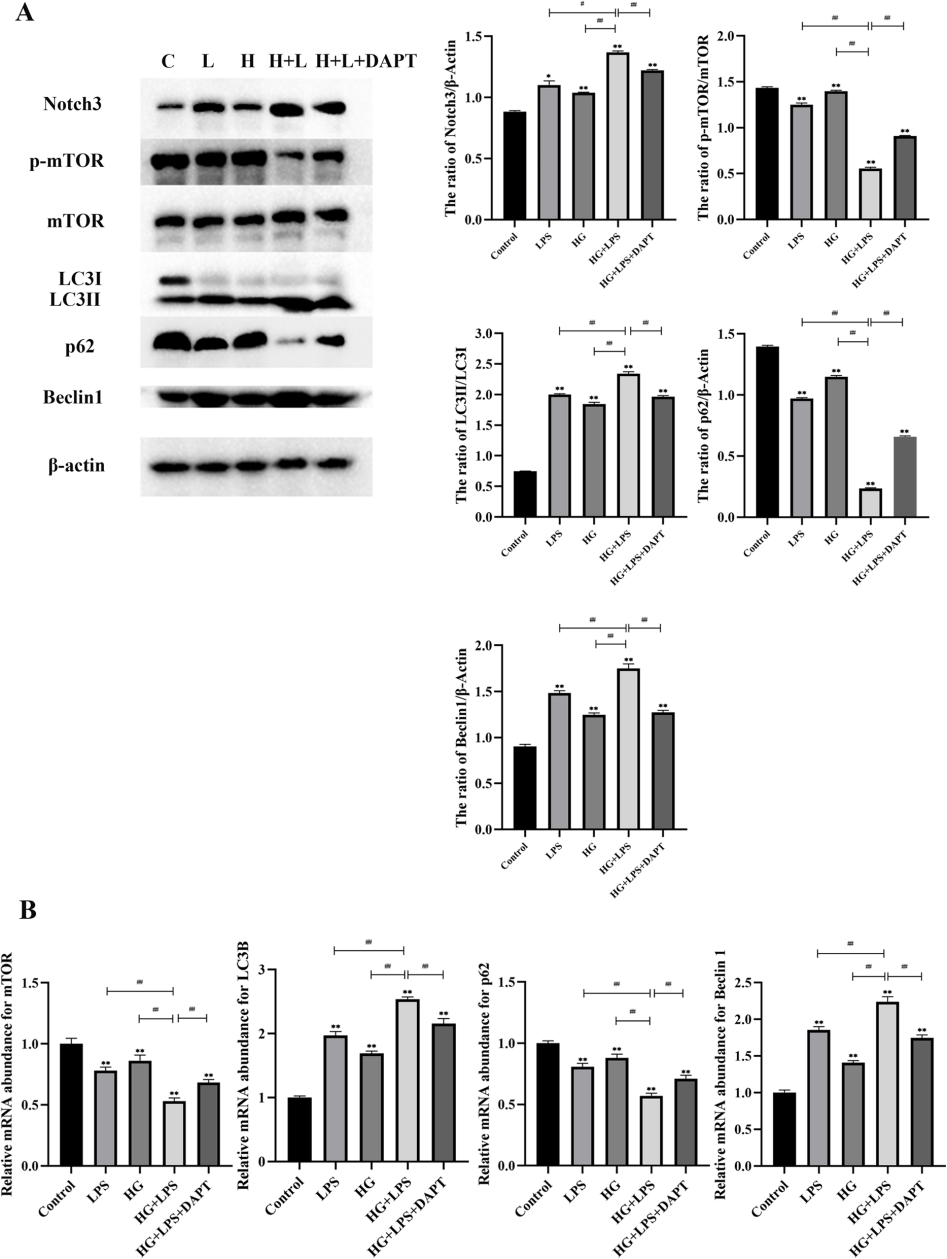
Co-treatment of HG and LPS induced autophagy by Notch3-mediated mTOR signaling pathways in MDBK cells. Note: MDBK cells are pretreated with 10 μM DAPT for 6 h, and then are stimulated with 5 μg/mL LPS and 25.5 mM glucose for another 24 h. **A** The protein level of Notch3, mTOR, p-mTOR, LC3-II/I, p62 and Beclin1. C: control; H: HG; L: LPS; H + L: HG + LPS; H + L + DAPT: HG + LPS + DAPT. **B** The mRNA expression level of Notch3, mTOR, LC3B, p62 and Beclin1. *0.01 < *P* < 0.05 and ** *P* < 0.01 compared with control group; #:0.01 < *P* < 0.05; ##: *P* < 0.01

To investigate the relationship between Notch3 and mTOR in autophagy induced by co-treatment of HG and LPS, MDBK cells were pretreated with DAPT, a specific Notch inhibitor. Firstly, the western blot showed that Notch3, LC3-II/I, and Beclin1 proteins were increased significantly (*P* < 0.01), whereas the p-mTOR and p62 proteins were decreased significantly (*P* < 0.01) in the HG + LPS treatment group compared with the single HG or LPS treatment group (Fig. 4A). Notch3, LC3-II/I, and Beclin1 proteins were decreased significantly (*P* < 0.01), and the p-mTOR and p62 proteins were increased significantly (*P* < 0.01) in the HG + LPS + DAPT treatment group compared with the HG + LPS treatment group (Fig. 4A). As shown in Fig. 4B, compared with the single HG or LPS treatment group, the LC3B and Beclin1 gene expression levels were increased significantly (*P* < 0.01), and the mTOR, and p62 gene expression levels were decreased significantly (*P* < 0.01) in the HG + LPS treatment group. Moreover, the LC3B and Beclin1 gene expression levels were decreased significantly (*P* < 0.01), whereas the mTOR and p62 gene expression levels were increased significantly (*P* < 0.01) in the HG + LPS + DAPT treatment group compared with the HG + LPS treatment group. In summary, DAPT reduced the autophagy induced by the co-treatment of LPS and HG.

**Fig. 5:**
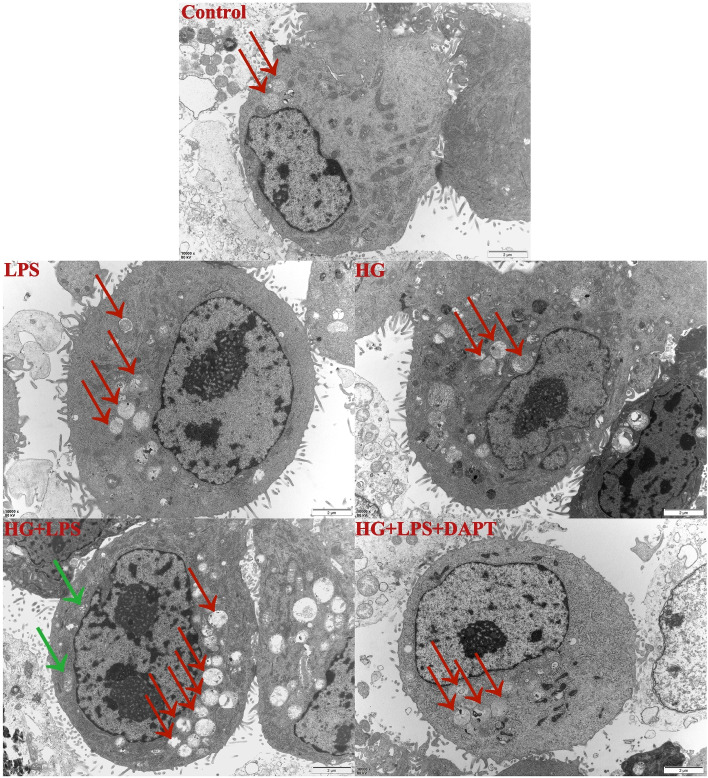
Autophagosomes under TEM. Note: MDBK cells are pretreated with 10 μM DAPT for 6 h, and then are stimulated with 5 μg/mL LPS and 25.5 mM glucose for another 24 h. Green arrows point to swollen mitochondria. TEM images of autophagic vacuoles (red arrow) were shown in MDBK cells from Control, HG, LPS, HG + LPS, and HG + LPS + DAPT treatment groups (scale bar: 2 μm)

Furthermore, the autophagosomes also were detected by TEM in the current study. The results showed that compared with control, the formation of autophagosomes were increased in the HG treatment group, LPS treatment group, and HG + LPS treatment group (Fig. 5). Moreover, compared with the HG + LPS treatment group, the HG + LPS + DAPT treatment group showed the reduced formation of autophagosomes as well as swelling of mitochondria (Fig. 5).

**Fig. 6:**
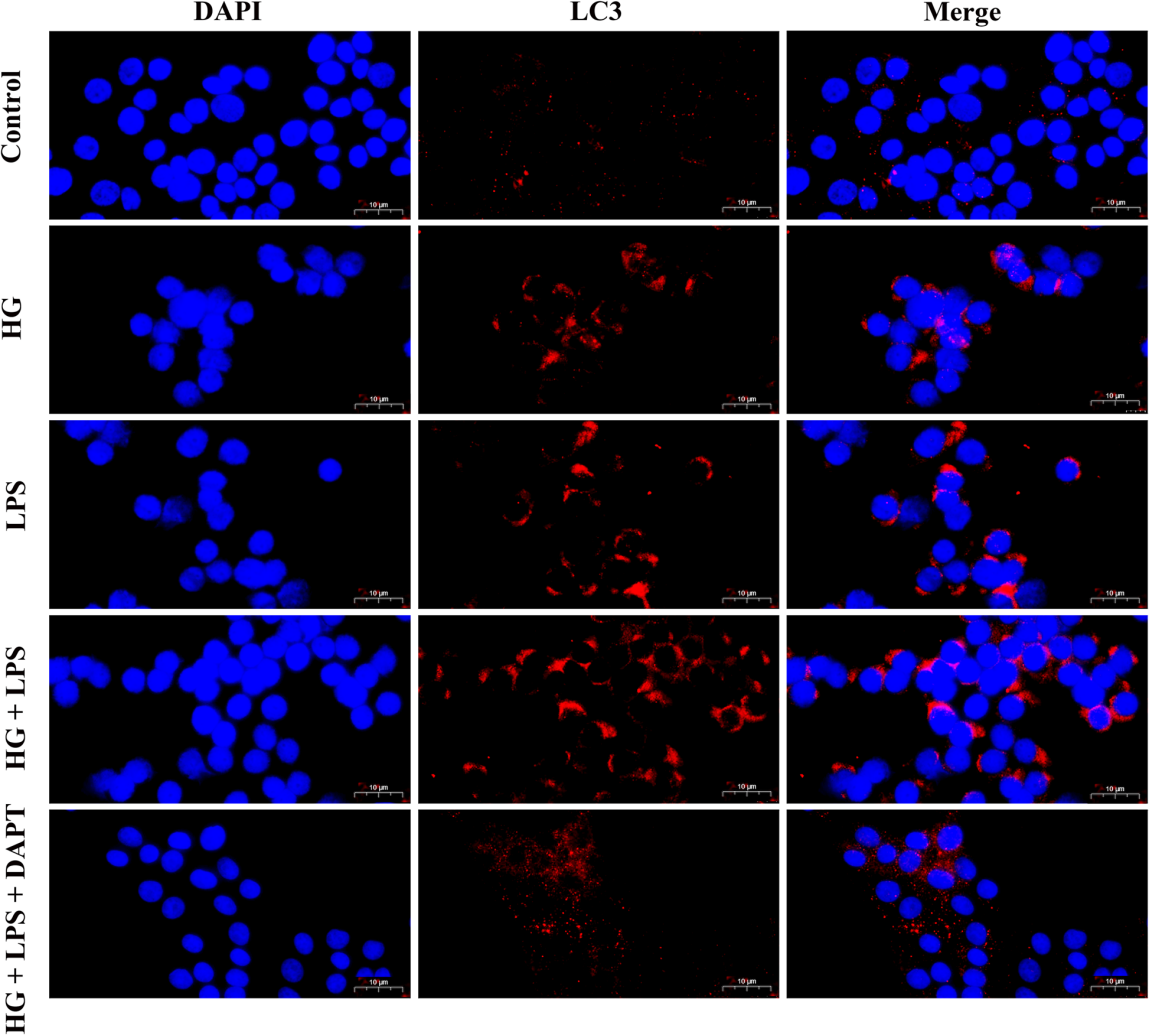
The fluorescence distribution of LC3. Note: MDBK cells are pretreated with 10 μM DAPT for 6 h, and then are stimulated with 5 μg/mL LPS and 25.5 mM glucose for another 24 h. LC3 was labeled with red fluorescence by Cy3, and the nucleus was labeled with blue fluorescence by DAPI. Immunofluorescence images of LC3 were shown in MDBK cells from Control, HG, LPS, HG + LPS, and HG + LPS + DAPT treatment groups. Scale bar, 10 µm

Similar results were also demonstrated in immunofluorescence assay. As presented in Fig. 6, compared with the control group, the intensity of red fluorescent labeled LC3 was increased in the HG treatment group, LPS treatment group, and HG + LPS treatment group. In addition, the intensity of red fluorescent labeled LC3 was decreased in HG + LPS + DAPT treatment group compared with the HG + LPS treatment group.

Overall, these findings demonstrated that the Notch3-mediated mTOR signaling pathway was involved in HG and LPS-induced autophagy in MDBK cells.

## Discussion

During transportation stress, severe kidney disease may occur together with respiratory diseases [45]. Hyperglycemia and gram-negative bacterial infection may be the main reasons why bovine is prone to severe kidney disease during transportation stress [1, 3, 46]. Hence, we established an in vitro model of MDBK cells treated with HG and LPS to investigate the pathogenic mechanism of high glucose and gram-negative bacteria on the kidney. In the current study, our results showed that the combined action of HG and LPS decreased MDBK cells viability.

Autophagy plays a central role in the survival and maintenance of cells by degrading organelles, proteins, and macromolecules in the cytoplasm and the circulation of breakdown products [47]. Moreover, autophagy plays a vital regulatory role in hyperglycemia-induced diabetes and LPS-induced sepsis. For example, autophagy was activated in type 2 diabetes mellitus erectile dysfunction rats [48]. LPS induced cardiomyocyte autophagy through miR-590-3p/AMPK/mTOR signaling pathway [49]. Recent studies have also found that autophagy plays an important role in the development of ruminant diseases [50]. However, whether autophagy is involved in renal injury in bovine during transport stress remains unclear. Firstly, we examined whether the combination treatment of HG and LPS induces autophagy in MDBK cells. In this study, the increase of LC3-II/LC3-I and Beclin1 while the decrease of p62 indicated that HG and/or LPS induce autophagy in MDBK cells. Similar to our results, Zhang et al. [51] have reported that LC3-II/LC3-I and Beclin1 protein increased, and p62 protein decreased in the H9C2 cardiomyocytes treated with HG. Kong et al. [52] have found that LC3 and Beclin1 protein were up-regulated in the human lung epithelial BEAS-2B cells treated with LPS. The process of autophagy is regulated by several evolutionarily-conserved genes called autophagy related genes (LC3, Beclin1, and p62) [53–56]. Meanwhile, the immunofluorescence results showed that the intensity of fluorescence increased in MDBK cells after the combination of HG and LPS.

Next, we investigated the mechanism of autophagy induced by the combination of HG and LPS. mTOR is a significant negative regulator of autophagy, conscientious for nutrient consumption, low energy, or oxidative stress [57]. In this experiment, the reduction of p-mTOR implied the capability of HG and LPS in inhibiting mTOR activities. Consistent with our results, Zhao et al. [58] have also proposed that HG exposure induced autophagy in H9c2 cells by inhibiting mTOR. It has been demonstrated that highly active Notch3 is generally found to be related to kidney injuries [59–61]. Besides, Notch3 also participate in autophagy through the interaction with mTOR, however, the relationship between them is still unclear. Previous studies have reported that mTOR is the major up-stream regulator of Notch3 activity [43], however, recent studies have found that the protein expression level of Notch3 is not affected when mTOR is inhibited [44]. In the current research, the increased levels of Notch3 implied that the Notch3 signaling pathway was involved in autophagy induced by HG and/or LPS in MDBK cells. Similar results were also observed in the study of Huang et al. [39], which reported that, the expression of Notch3 was significantly increased in the renal tubular epithelial cells of 18 patients with obstructive nephropathy. Next, the relationship between Notch3 and mTOR was investigated in this study. From our study, co-treatment with the Notch3 inhibitor (DAPT) could reverse the reduction of p-mTOR protein expression, indicating that Notch3 was the major up-stream regulator of mTOR. These results were consistent with previous findings by Ivanovska et al. [44]. Meanwhile, ultrastructure observation and LC3 immunofluorescence staining results showed that Notch3 inhibition could abolish the autophagy induced by HG and LPS.

## Conclusion

**Fig. 7:**
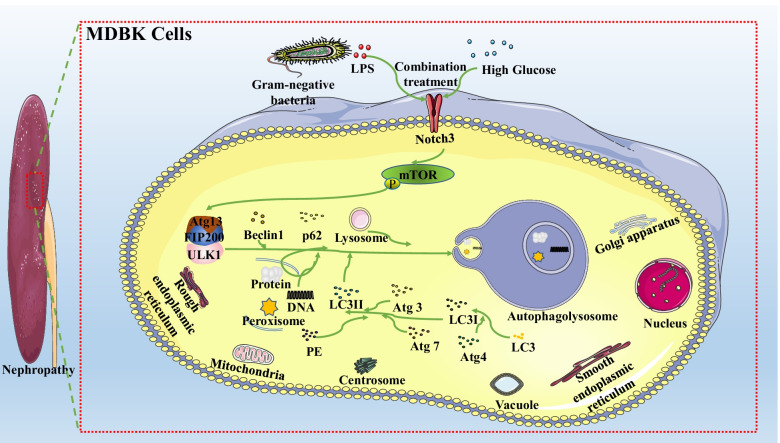
Schematic diagram for the proposed autophagy signaling pathways in MDBK cells regulated by the combination effect of HG and LPS. In HG and LPS-stimulated MDBK cells, the Notch3-mediated mTOR signaling pathway is activated, resulting in the activation of autophagy-related proteins and genes. Abbreviations HG: High glucose; LPS: Lipopolysaccharide; MDBK: Madin-darby bovine kidney; mTOR: Mammalian target of rapamycin; DMEM: Dulbecco’s Modified Eagle Medium; CCK-8: Cell counting kit-8; qRT-PCR: Quantitative real-time PCR; TEM: Transmission electron microscopy

In conclusion, this study demonstrated that the combination of HG and LPS could induce autophagy in MDBK cells, and the Notch3-mediated mTOR signaling pathway was involved in HG and LPS-induced autophagy, as summarized in Fig. 7. This study provides a theoretical basis for the pathogenesis of nephropathy caused by the combination of HG and LPS, hence will help in the development of therapeutic strategies to resolve kidney toxicity associated with bacterial infection under hyperglycemia conditions in cattle and bacterial infection under diabetic nephropathy. However, the role of autophagy in HG and LPS-induced renal toxicity needs to be further explored.

## Supplementary Information

**Additional file 1: Figure S1.** Uncropped blots images displayed in the context.

**Additional file 2: Figure S2.** The effects of HG combined with LPS on autophagic flux. MDBK cells were pretreated with CQ (10 μM) for 6h, and then stimulated with 5 μg/mL LPS and 25.5 mM glucose for another 24 h, and the protein expression level of LC3 was detected by the Western bolt. * *p* < 0.05 and ** *p* < 0.01 compared with control group.

## Data Availability

The raw data is available from the corresponding author upon reasonable request.
