# Supplementary material for: The combination of high glucose and LPS induces autophagy in bovine kidney epithelial cells via the Notch3/mTOR signaling pathway
Source: BMC Vet Res. 2022 Aug 11;18:307. doi: 10.1186/s12917-022-03395-1 (PMC9367163; doi:10.1186/s12917-022-03395-1)

Additional file 1 Figure S1: Uncropped blots images displayed in the context

WB original figure corresponds to the figure annotation in the manuscript.

Figure 1 Co-treatment of HG and LPS induced autophagy in MDBK cells.

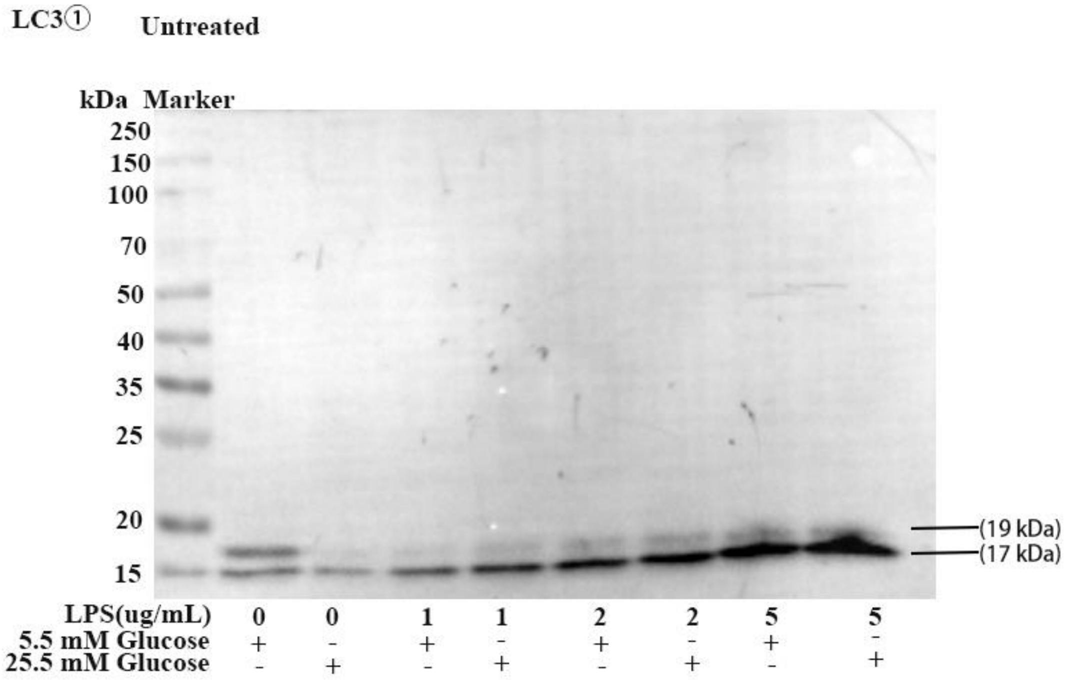

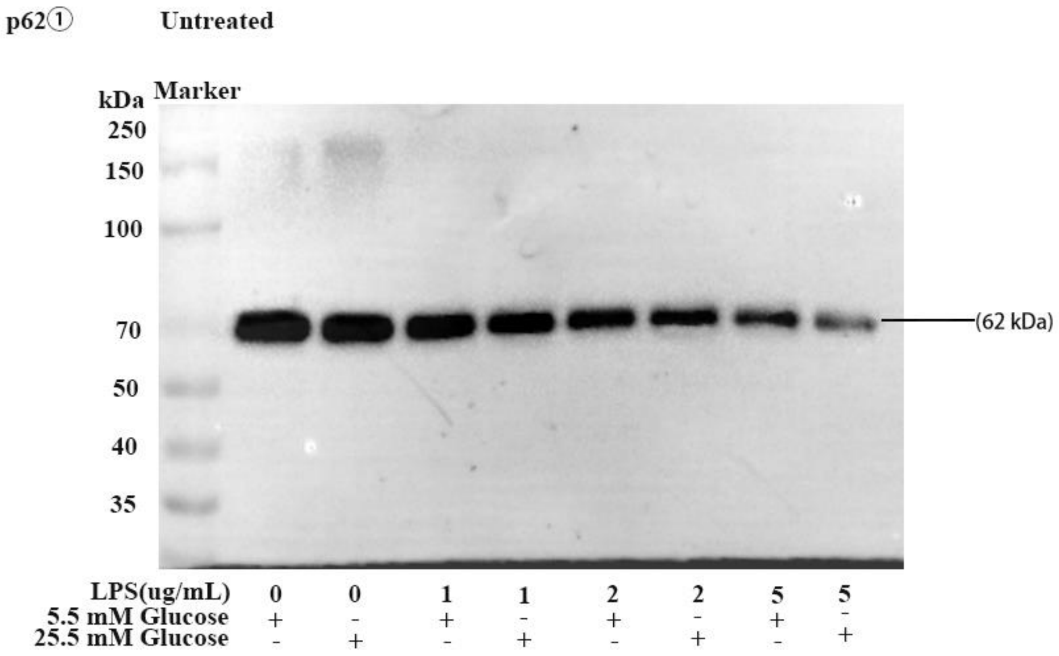

Beclin 1① Untreated

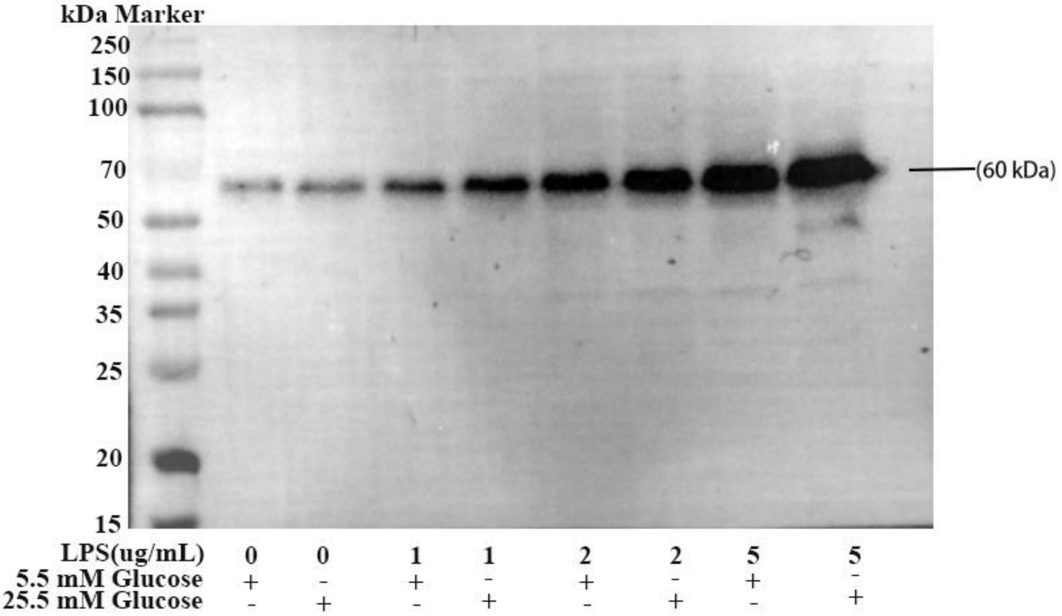

β-actin① Untreated

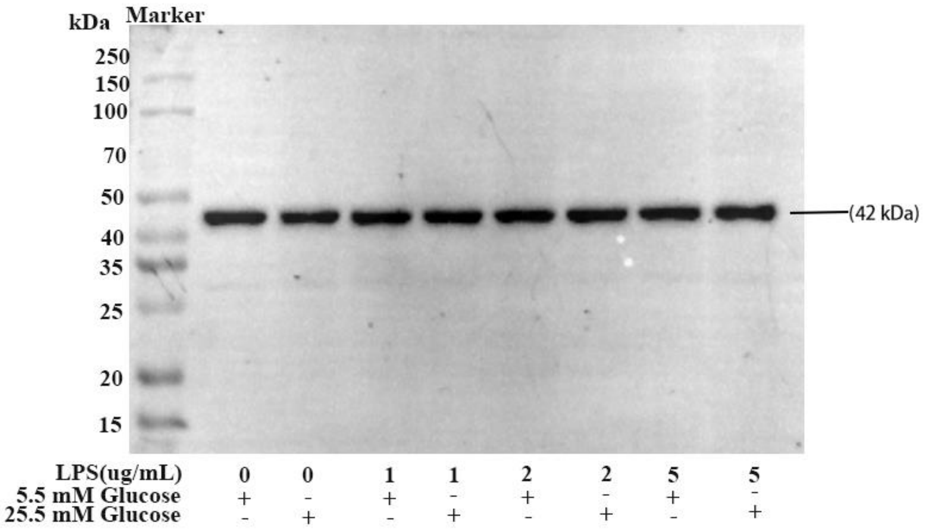

Figure 3 Co-treatment of HG and LPS induced Notch3 and mTOR signaling pathways in MDBK cells.

Notch3② Untreated

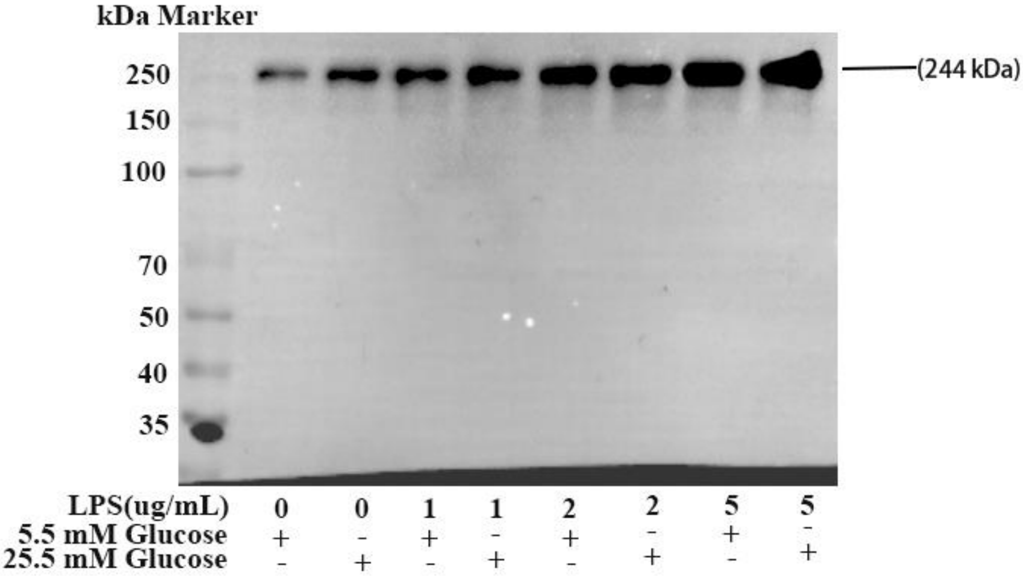

p-mTOR② Untreated

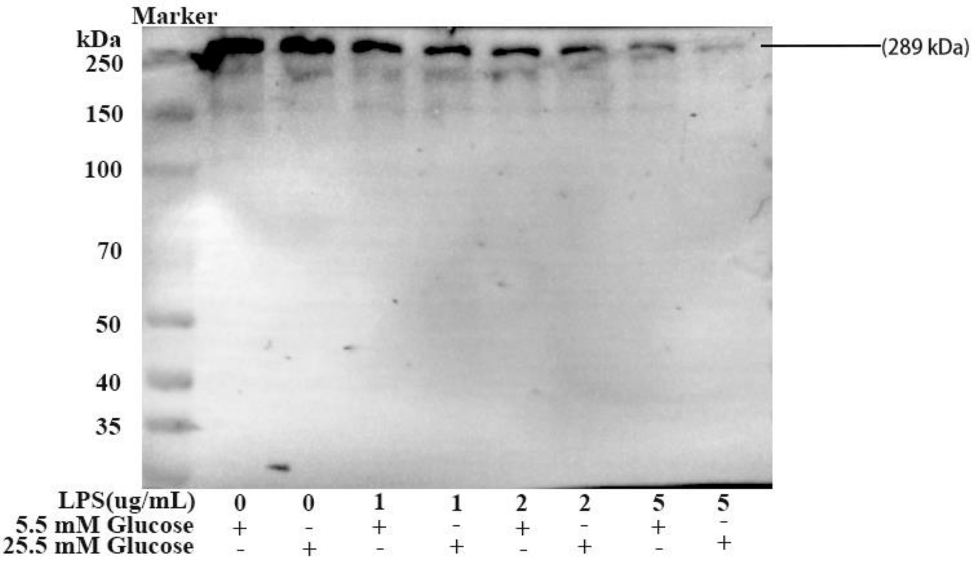

mTOR② Untreated

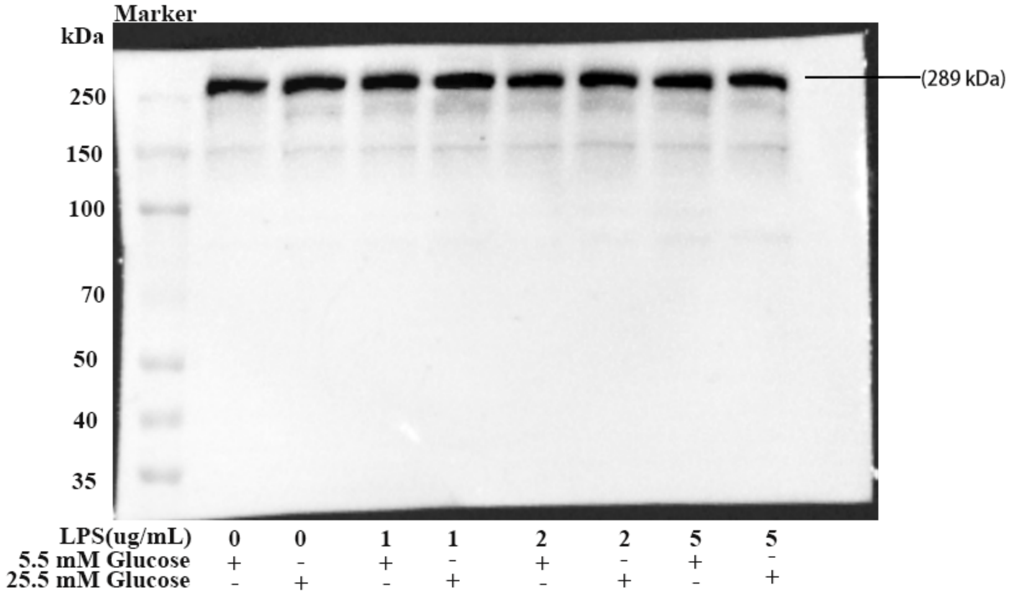

β-actin② Untreated

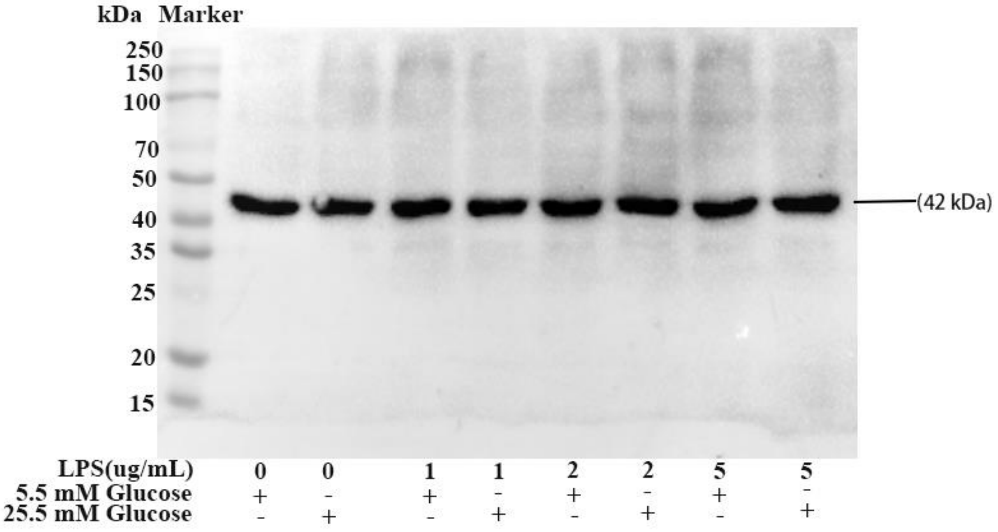

Figure 4 Co-treatment of HG and LPS induced autophagy by Notch3-mediated mTOR signaling pathways in MDBK cells.

Notch3③ Untreated

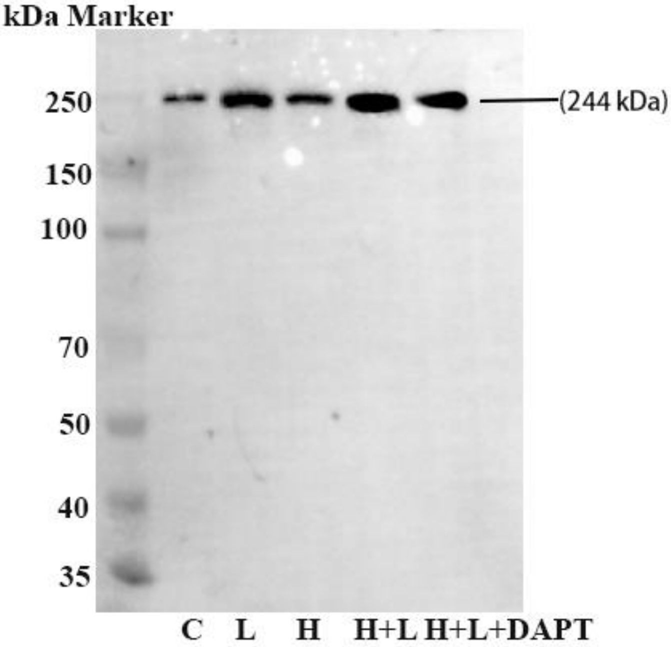

p-mTOR③ Untreated

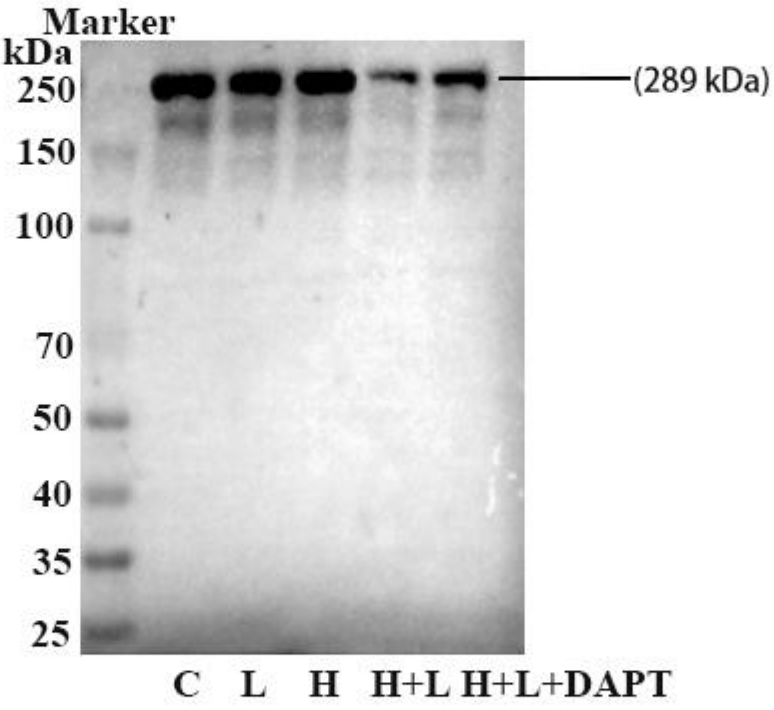

mTOR③ Untreated

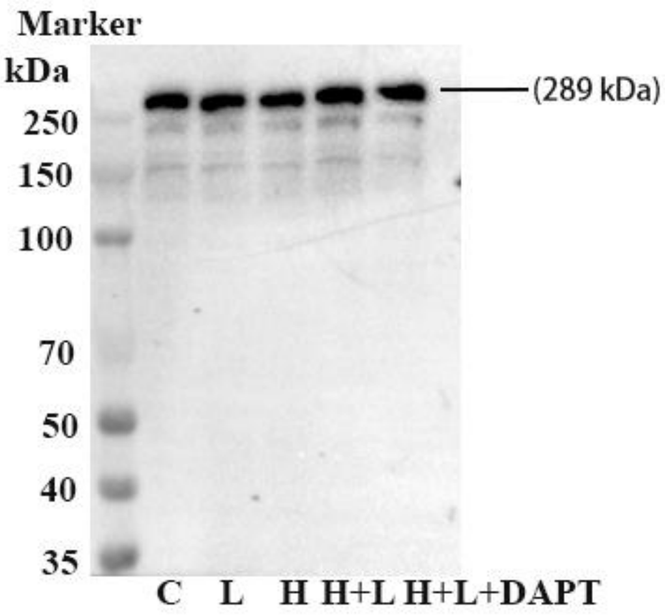

LC3③ Untreated

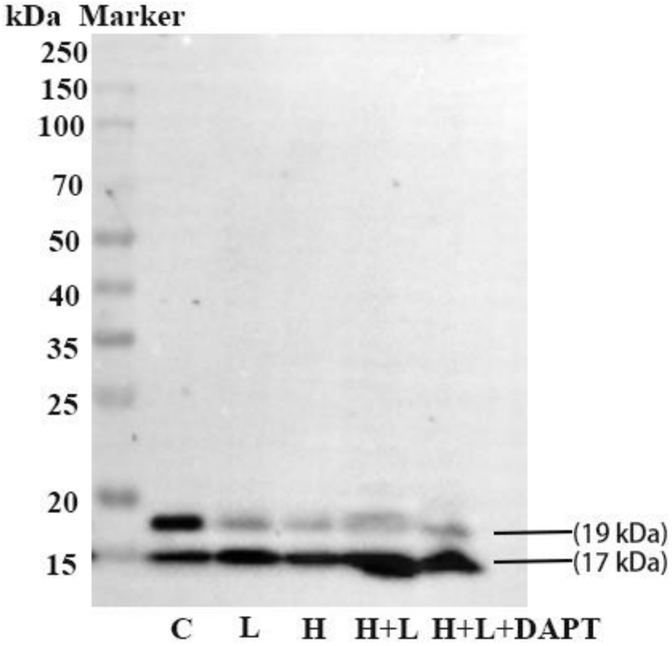

p62③ Untreated

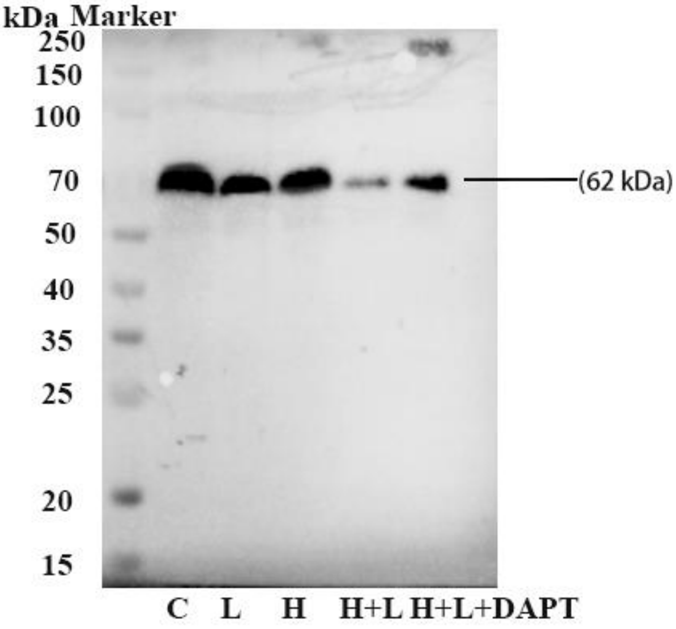

Beclin 1③ Untreated

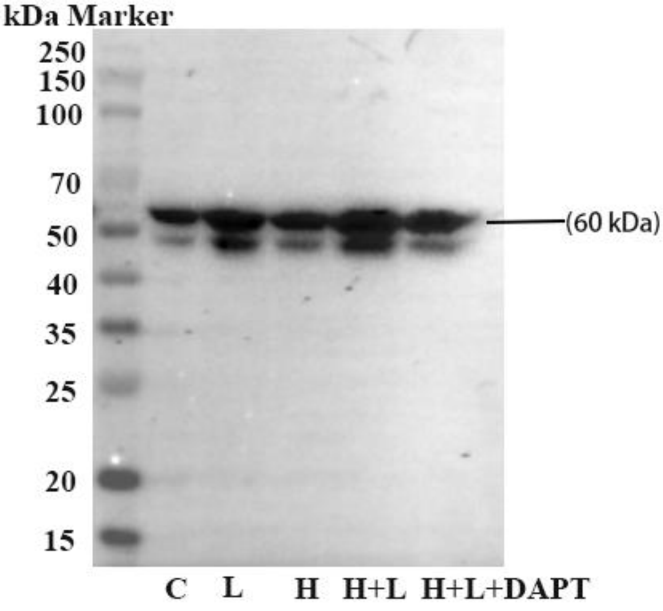

β-actin③ Untreated

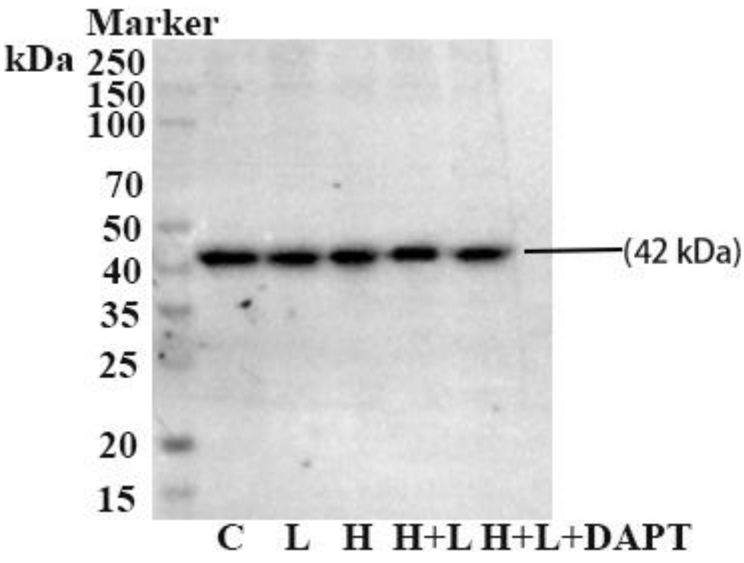

Figure S2: The effects of HG combined with LPS on autophagic flux.

LC3④ Untreated

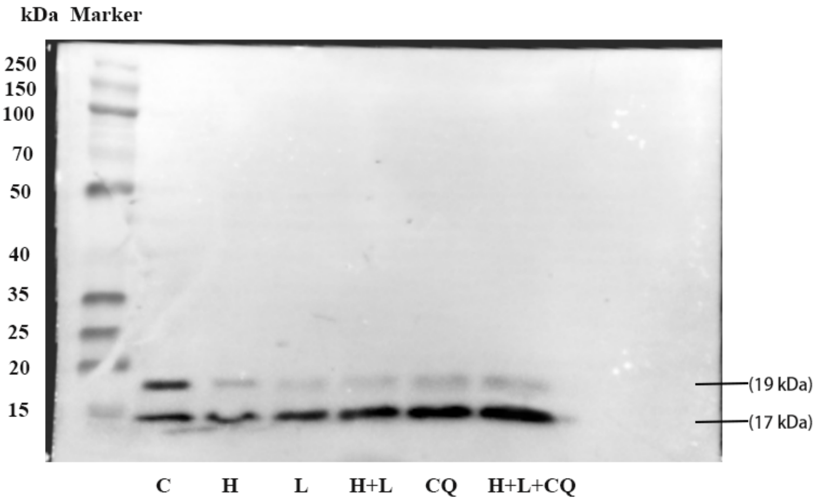

β-actin④ **Untreated**

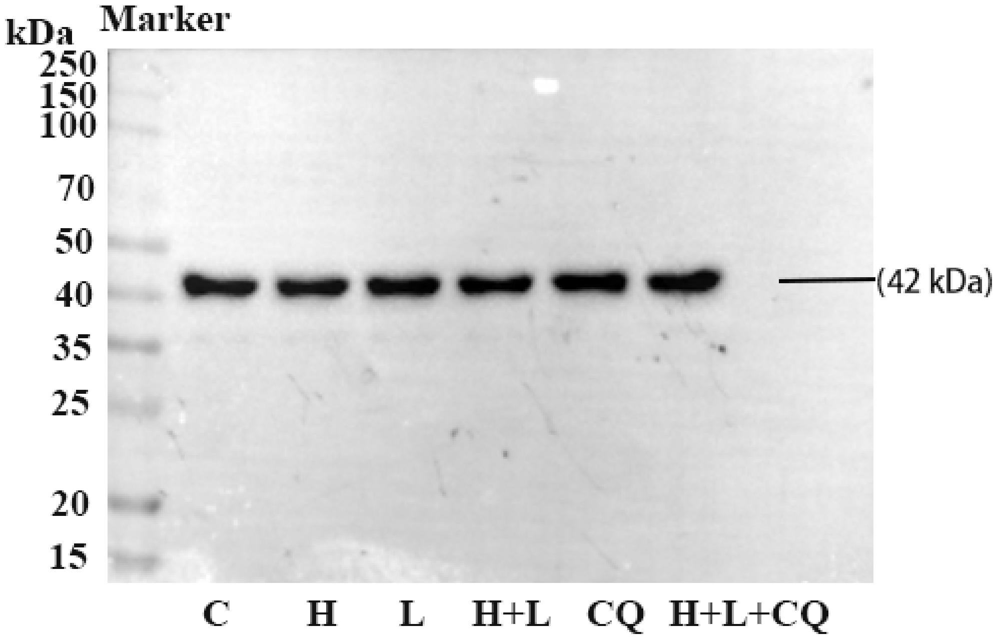

Supplement: Supplementary file 1 — Additional file 1: Figure S1. Uncropped blots images displayed in the context. [file 12917_2022_3395_MOESM1_ESM.pdf]
