# Supplementary material for: The combination of high glucose and LPS induces autophagy in bovine kidney epithelial cells via the Notch3/mTOR signaling pathway
Source: BMC Vet Res. 2022 Aug 11;18:307. doi: 10.1186/s12917-022-03395-1 (PMC9367163; doi:10.1186/s12917-022-03395-1)

Additional file 2 Figure S2: Uncropped blots images displayed in the context

Figure S2: The effects of HG combined with LPS on autophagic flux.

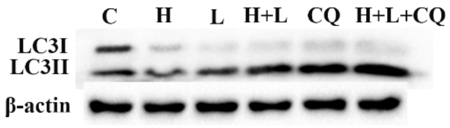

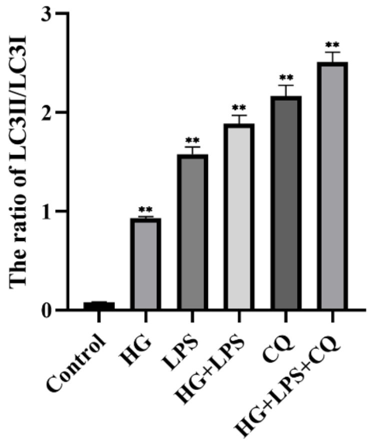

Supplement: Supplementary file 2 — Additional file 2: Figure S2. The effects of HG combined with LPS on autophagic flux. MDBK cells were pretreated with CQ (10 μM) for 6h, and then stimulated with 5 μg/mL LPS and 25.5 mM glucose for another 24 h, and the protein expression level of LC3 was detected by the Western bolt. * p < 0.05 and ** p < 0.01 compared with control group. [file 12917_2022_3395_MOESM2_ESM.pdf]
